# The value of allied health professional research engagement on healthcare performance: a systematic review

**DOI:** 10.1186/s12913-023-09555-9

**Published:** 2023-07-18

**Authors:** S. Chalmers, J. Hill, L. Connell, S. Ackerley, A. Kulkarni, H. Roddam

**Affiliations:** 1 University of Central Lancashire; Allied Health Research Unit, School of Health Sciences, University of Central Lancashire, Fylde Rd, Preston, PR1 2HE UK; 2 Bolton NHS Foundation Trust, Minerva Road, Farnworth, Bolton, Greater Manchester, BL4 0JR UK; 3 University of Central Lancashire; Synthesis, Economic Evaluation and Decision Science (SEEDS) Group, University of Central Lancashire, Fylde Rd, Preston, PR1 2HE UK; 4 East Lancashire Hospitals NHS Trust, Burnley, BB10 2PQ UK; 5 Royal College of Speech & Language Therapists, 2-3 White Hart Yard, London, SE1 1NX UK; 6 Subject Matter Expert for AHP Research, Health Education England, Manchester, UK

**Keywords:** Research engagement, Allied health, Healthcare performance, Allied Health Professionals (AHPs)

## Abstract

**Background:**

Existing evidence suggests that clinician and organisation engagement in research can improve healthcare performance. With the increase in allied health professional (AHP) research activity, it is imperative for healthcare organisations, clinicians, managers, and leaders to understand research engagement specifically within allied health fields. This systematic review aims to examine the value of research engagement by allied health professionals and organisations on healthcare performance.

**Methods:**

This systematic review had a two-stage search strategy. Firstly, the papers from a previous systematic review examining the effect of research engagement in healthcare were screened to identify papers published pre-2012. Secondly, a multi-database search was used to conduct a re-focused update of the previous review, focusing specifically on allied health to identify publications from 2012–2021. Studies which examined the value of allied health research engagement on healthcare performance were included. All stages of the review were conducted by two reviewers independently. Each study was assessed using the appropriate Joanna Briggs Institute critical appraisal tool. A narrative synthesis was completed to analyse the similarities and differences between and within the different study types.

**Results:**

Twenty-two studies were included, comprising of mixed research designs, of which six were ranked as high importance. The findings indicated that AHP research engagement appears related to positive findings in improvements to processes of care. The review also identified the most common mechanisms which may link research engagement with these improvements.

**Discussion:**

This landmark systematic review and narrative synthesis suggests value in AHP research engagement in terms of both processes of care and more tentatively, of healthcare outcomes. While caution is required because of the lack of robust research studies, overall the findings support the agenda for growing AHP research. Recommendations are made to improve transparent reporting of AHP research engagement and to contribute essential evidence of the value of AHP research engagement.

**Trial registration:**

This systematic review protocol was registered with the international prospective register of systematic reviews, PROSPERO (registration number CRD42021253461).

**Supplementary Information:**

The online version contains supplementary material available at 10.1186/s12913-023-09555-9.

## Introduction

### Background

Within the United Kingdom (UK), national policy drivers aim to support the research engagement of healthcare professionals. The National Health Service (NHS) Long Term Plan [1] specifically identifies that research and innovation is fundamental to driving future health improvement and the Department of Health and Social Care’s strategy for the future of UK clinical research states that a ‘sustainable and supported (health) research workforce’ is fundamental to achieving such impact [2]. Given this prominent focus at a UK-wide policy level, it is imperative to understand the value of healthcare professionals’ engagement in and with research. This is a highly topical universal agenda that has generated the recent publication of position statements from worldwide health and care organisations (including, but not limited to the Australian Allied Health Professors’ “The Value of Allied Health Research” [3]; the Chief Nursing Officer for England’s strategic plan for research [4]; and the Royal College of Physicians’ “Making research everybody’s business” [5]).

Previous research [6, 7] has systematically reviewed whether engagement of clinicians and organisations in research improves healthcare performance. This work also explored the possible mechanisms at play; defined as the levers that instigate a relationship between research engagement activities and improved health care, for example improvements in infrastructure, staff training, linkage and exchange between organisations, and research networks [6]. Within their analysis, the researchers took ‘engagement *in* research’ to mean a “deliberate set of intellectual and practical activities undertaken by healthcare staff and organisations…” (p2) [6]. This contrasted with a broader definition of research engagement to include ‘engagement *with* research’, meaning “less substantial involvement at individual and team level related more to receiving and transmitting the findings of research”(p3) [6].^1^ Health care performance was conceptualised to include a wide range of measures including “measures of clinical process, health outcomes, access, efficiency, productivity, and employee variables”(p3, [6]). By using a developed matrix which analysed the level of engagement, impact, direction of findings, and outcomes [7], the review concluded that when clinicians and organisations engage *in* research, it is likely that healthcare performance improves. It is worth noting that the studies (identified from the 2012 search strategy) were predominately set within the context of medicine, surgery, or nursing. Whilst some of the studies included mixed populations of healthcare professionals, only one paper specifically referenced the involvement of Allied Health Professionals (physical therapists) [8].

Allied Health Professionals (AHPs) are the third largest clinical workforce in the NHS. The definition of AHP varies internationally but in England, the AHP workforce is comprised of “… 14 professions^2^ working across the spectrum of health and care … from primary to specialist care provision”(p5) [9]. It is also acknowledged that AHPs are involved in research, research mentorship and supervision, charities, business, and industry. Over the past decade, research engagement among AHPs has gained momentum as it has been acknowledged that along with nurses and midwives, AHPs could become central to innovative patient care as clinical academics in the years ahead [10]. This development is recognised through the expanding literature in allied health research engagement strategies, activity, funding, and capacity, exemplified by initiatives such as the creation of the National Institute of Health Research (NIHR) Integrated Clinical Academic pathway [10]. The agenda to accelerate the growth of AHP research has been published in Health Education England’s (HEE) AHP Research and Innovation Strategy for England 2022 [11], which identifies that “securing and sustaining excellence in research and innovation for the Allied Health workforce is (now) a global priority agenda” (p5). Furthermore, the AHPs Strategy for England: AHPs Deliver [9] defines ‘research, evaluation and innovation’ as one of its four enhanced foundations and states the “expectation is that AHPs commit to research, innovation and evaluation (and)…implementation initiatives across these significant agendas will support enhanced engagement and impact” [11]. Given this ambition it is imperative for healthcare organisations, AHP clinicians, managers, and leaders to understand the value of research engagement on healthcare performance within these specific AHP disciplines.

In 2019, a qualitative systematic review exploring a broad range of impacts of clinical academic activity by healthcare professionals outside of medicine included two studies exclusively involving AHPs [12]. The paper identified impacts which mapped to seven themes. For example, impacts for patients demonstrated the beneficial changes to service provision that arose from clinical academic activity and improved access to evidence-based healthcare. Impacts on service provision highlighted that clinical academic activity was regarded as beneficial because it resulted in enhanced care delivery and pathways. Other themes included impact to the clinical academic, research profile, and culture and capacity. Despite some of these themes broadly aligning to the processes of care and health outcomes previously identified [6], the methodology used and the small number of studies focusing on AHPs mean the question remains as to the value of research engagement, specifically by AHPs, on healthcare performance.

This systematic review provides a focused exploration of the value^3^ of AHP research engagement on healthcare performance, by drawing from the methodology utilised in the previous broader review [7] and utilising the same definitions of the core concepts. This will not only support AHP clinicians to carefully consider the potential value of their research engagement, it will also enable health organisations, clinicians, managers, leaders, and policy makers to make informed decisions about AHP services, policy, education, and workforce.

### Objectives

1. To systematically review published literature exploring the value of research engagement by AHP clinicians and organisations on healthcare performance.
2. To identify mechanisms that may instigate a relationship between research engagement activities and improved healthcare performance.

## Methods

### Search strategy

This review was undertaken and reported in accordance with a previously published protocol [13] and the PRISMA reporting guidelines [14]. Firstly, full paper screening was undertaken independently by two reviewers of all included studies from the previous systematic review [6, 7] to identify any relevant studies published pre-2012. Secondly, a multi-database search was carried out on Medline, Embase, Health Management and Policy Database (HMIC), PsychINFO, The Cumulative Index to Nursing and Allied Health Literature (CINAHL), and OpenGrey from 2012 – June 2021. The same search strategy which was used in the previous review [6, 7] was utilised with additional terms for AHPs (see Additional file 1 for example search strategy). Additionally, all included studies’ reference lists were screened for eligible studies.

### Inclusion and exclusion criteria

The study protocol [13] was adopted from a previous study [6] conducted by Boaz et al. but with a specific focus on AHPs, as opposed to all healthcare professionals. However, following preliminary searches of the literature, it was recognised that this approach would not allow us to meet our original objective in the protocol which focused on effectiveness because of the paucity of research specifically focused on AHPs fitting this strict criteria.

#### ‘Value’

Amendments were made to broaden the inclusion criteria and take a more pragmatic approach with a focus on value rather than effectiveness as stipulated in the original protocol [13]. See Table 1 for the broadened study inclusion criteria compared to the protocol. The broadened focus on value was used to illustrate the concept of positive improvement (where research engagement showed improved healthcare performance), the type of outcome, and the type of impact. This was based on the work of Hanney et al. in their development of the theoretically driven matrix [7]. This analysis is detailed in Table 3.

**Table 1:** Broadened inclusion criteria in comparison to the protocol, with examples of concepts

Variable	Protocol inclusion criteria	Broadened inclusion criteria
Population	Studies solely including allied health professionals	Studies including a mixed population of healthcare professionals; encompassing a partial sample of AHPs, stated explicitly or implied by the clinical context. Any of the registered AHPs^2^ were included (including their teams and organisations) which work within health, social and/or educational settings, as specified in the AHP Research and Innovation Strategy for England 2022 [11]
Intervention	Studies making explicit reference to engagement *in* research. This incorporated studies focusing on AHPs and organisations that were directly involved in: (a) agenda setting, (b) conducting research, (c) action research, or (d) research networks where the engagement in research is noted	Studies addressing engagement *in* research, as well as engagement *with* research, which also included evidence-based clinical professional develop, evidence-based practice, implementation efforts, critical appraisal, research utilisation, and adoption of research in policy making or clinical guidelines
Comparison	Studies with or without a comparator	No amendment
Outcomes	The primary outcome of this review was healthcare performance (processes of care or health outcomes) assessed pre- and post-research engagement	No amendment
Study type	Effectiveness studies: randomised control trials, repeated measured or quasi-experimental studies. Mixed method studies were considered where an effectiveness component was included in the study and this directly related to the outcome of healthcare performance	Any primary research study type with reference to research engagement

#### Research engagement terminology

Terminology to describe research engagement is problematic [15] and include phrases such as ‘engagement *in* research’ and ‘engagement *with* research’*.* These are often used interchangeably despite efforts made by Hanney et al. to distinctly define these [7]. The broadened inclusion criteria uses the term ‘research engagement’ as an umbrella term referring to the inclusion of both engagement *in* and *with* research (see Table 1 for details). As specified in the aims and objectives, the focus in this review is on research engagement by AHPs, as opposed to alternative interpretations such as patient/service-user or public research engagement or involvement.

#### Indirectness classification

Due to the amendments made, it was important to demonstrate the applicability of included studies to the protocol inclusion criteria (population, intervention, and study design variables) through a classification of indirectness [16]. All included studies were classified using a 1-to-10-point Likert scale (Table 2) with a score of 10 meaning that it would fully meet the inclusion criteria in the protocol for that particular variable. The values between the 10-point scale were determined through discussion with the research team, GRADE recommendations of indirectness [16], and based upon the principles in Table 2.

**Table 2:**
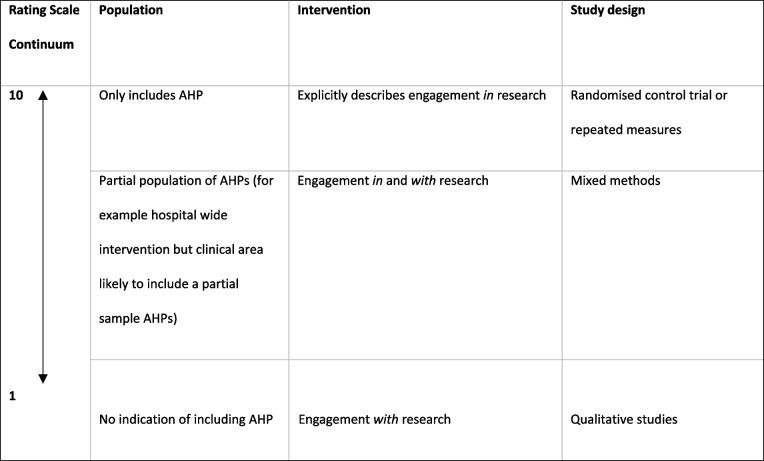
10-point Likert scale to judge the relevancy of each study to the inclusion criteria set out in the protocol

### Selection process

Duplication removal was undertaken using both EndNote and Rayyan [17]. References were uploaded and managed on the Rayyan web database to facilitate the screening process. An initial screening of 10 abstracts and titles was undertaken independently by all reviewers to ensure consistency of screening. All titles and abstracts were reviewed by two independent assessors (SC and SA, LC, AK, or HR). Full papers were screened independently by two reviewers (SC, JH). For both title, abstract, and full paper screening, disagreements were resolved by discussion. If consensus was not achieved, arbitration was carried out by a third reviewer. All reasons for selection or rejection of full papers were recorded.

### Data collection

Two reviewers independently completed data extraction on a pre-piloted data extraction form (SC, JH, SA, LC, AK, HR) (see Additional file 2 for an example data extraction form). The data extraction table was stored as an Excel spreadsheet. Disagreements and inconsistencies were resolved by discussion and where consensus could not be achieved arbitration was carried out by the research team.

All included studies were analysed using the theoretically driven matrix developed [7] by Hanney et al. to characterise the dimensions in which research engagement might have led to healthcare performance outcomes. The matrix was developed through an iterative process which evaluated existing reviews and theories [7]. The matrix enabled extraction of salient information across the following dimensions: degree of intentionality, level of study engagement, impact, findings, and outcomes (see Table 3 for the full description of each dimension). The importance of each paper to the review was assessed and completed by two independent reviewers (SC, JH, SA, LC, AK, HR). The quality assessment and study type were the most important aspects to judge importance, followed by the indirectness of population and intervention in relation to the inclusion criteria. For example, a study which is of experimental design with a relevant population but classified as engagement with research would be more important when establishing the value of research engagement; compared with a qualitative study with a mixed population with engagement in and with research. Additional data items that were sought recorded: paper title, authors, year, country, allied health profession, organisation, clinical setting, study design, research question, nature of research engagement activity (intervention), methods, outcome measures, and quality assessment.

**Table 3:** Data analysis dimensions identified in the theoretically driven matrix [6, 7]

Data item	Category	Key	Full definition
**Level of study engagement**	Organisational level	O	Level of engagement discussed either at organisational or clinician level
	Clinician level	C	
**Impact**	Specific	S	“Refers to those who had engaged in research being more willing and/or able to provide evidence-based care that was related to the specific findings of the research in which they were engaged.”
	Broad	B	“Refers to those who had engaged in research being more willing and/or able to provide evidence-based care that was based on relevant research conducted anywhere and, and that was not related to the specific findings of the research in which they were engaged.”
**Findings**	Positive	+	Where the findings of the paper were positive or negative in relation to the review objective. Studies were classified to be positive if they showed research engagement did improve healthcare performance, and negative if not. e.g. All healthcare performance outcomes reported within the study were positive. With such outcomes being participation in local recommendation development and research involvement from baseline to end of study. When the paper reported mixed findings, this would be a mixture of positive and negative findings
	Negative	-	
	Mixed	M	
	Mixed-positive	M +	
	Mixed-negative	M-	
**Improvement identified**	Processes of care	P	The nature of the healthcare performance improvement identified in the paper
	Health outcomes	HO	
**Importance**	High	1	Integrated assessment based on firstly the quality assessment and study type, and secondly the relevancy of population and intervention to the review question
	Low	2	

### Quality Assessment

Quality appraisal was carried out by two independent reviewers with arbitration by a third reviewer (SC, JH, SA, LC, AK, HR). The diversity of methods used in the studies meant that one quality appraisal tool could not be applied universally. The research team selected the most appropriate Joanna Briggs Institute critical appraisal tool [18] and the Mixed Methods Appraisal Tool [19] based on the design of each included study.

### Synthesis methods

The diversity of study designs among the included studies made it impossible to conduct a meta-analysis of the results. There was no minimum number of studies required for the synthesis, and exclusion was not made following the quality assessment due to the overall paucity of research in this area. A narrative synthesis was completed to analyse the similarities and differences between and within the different study types which reported positive findings, and compare the level of relevance to the population and intervention in relation to the inclusion criteria. The value of research engagement was assessed narratively by describing the positive studies (where research engagement improved healthcare performance) and were grouped around the perceived importance (triangulation of indirectness of study design, population, and intervention [16]) of each individual study.

### Subgroup analysis

Each paper included within this review was additionally examined for any factors that the study authors proposed as potential components of the improvement in healthcare performance. Hanney et al. developed a taxonomy of the various mechanisms and sub-mechanisms through which outcomes may be superior in research-active settings [7]. The 12 mechanisms identified and described in the previous review were used in a pre-defined coding framework (see Additional file 3). The mechanisms were described regarding the most common to least common. Due to the majority number of studies which reported positive findings and the wide variation in the indirectness classification, it was judged that vote counting [20] of positive studies for each mechanism would not be appropriate.

## Results

### Study selection

**Fig. 1:**
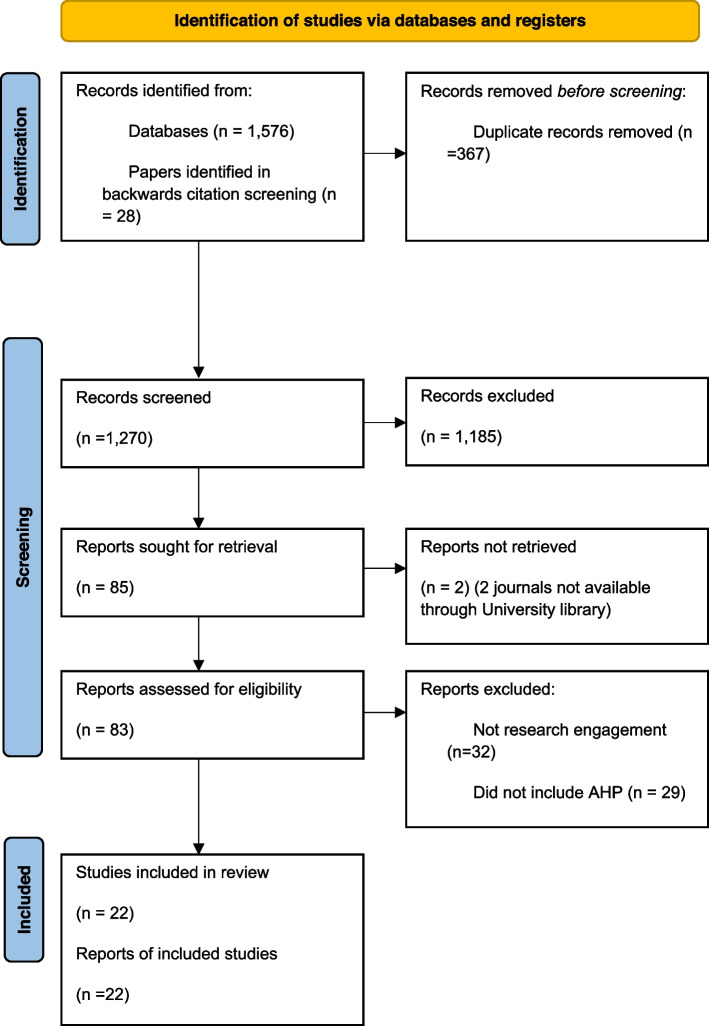
Identification of studies via databases and registers

After duplicate removal, 1,209 papers were identified from the search strategy. An additional 28 papers were identified through backwards citation screening and 33 papers were included from the previous review [6]. Subsequently, 1,270 papers were identified for screening. After title and abstract screening, 85 papers were retrieved for full paper screening. There was a 98.24% agreement between the first and second reviewers (k = 0.67). Two papers were unable to be retrieved, and 61 papers were excluded of which 32 papers had an incorrect intervention and 29 papers did not include AHP. This resulted in 22 studies being eligible to be included in this review (See Fig. 1 for PRISMA Flow Diagram [14, 21]). Out of the 22 studies, 7 were identified from the previous review, 14 were identified through the primary search strategy [22–34], and one study was identified through screening of included studies’ reference lists [35].

### Study characteristics

**Fig. 2:**
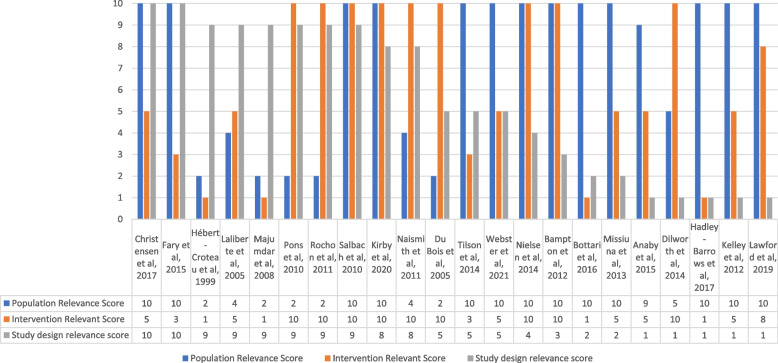
Relevancy of all included studies to the inclusion criteria set out in the protocol. Key: Population: 1 = No indication of including AHP, 10 = Only includes AHP; Intervention: 1 = *Engagement with research,* 10 = Explicitly describes *engagement in research*; Study design: 1 = Qualitative studies, 10 = RCT or repeated measures

Studies were conducted in seven different countries: five in Canada [8, 22, 23, 31, 36], five in Australia [25, 26, 30, 32, 35], four in the United States of America [24, 33, 37, 38], four in the United Kingdom [27, 29, 34, 39], two in Germany [40, 41], one in Spain [42], and one in Ireland [28]. These studies took place from 1999 to 2021. The studies were conducted in a wide range of clinical areas with seven studies in Oncology [25, 34, 36, 37, 39–41], four studies in Musculoskeletal Physiotherapy [26, 27, 30, 32], three studies in Neurology [8, 23, 35], three studies in Paediatrics [22, 24, 31], two studies in Cardiology [38, 42], two studies in multiple clinical areas [28, 33], and one study in Respiratory [29]. Where studies included a single discipline, the professional disciplines cited were: Physiotherapists (*n* = 8) [8, 24, 26, 27, 30, 32, 33, 35] followed by Occupational Therapists (n = 3) [23, 28, 31], Paramedics (*n* = 1) [29], and Radiographers (*n* = 1) [34]. The remaining nine studies included only partial sample populations of AHPs. Included within these nine studies was a combination of occupational therapists, physiotherapists, and a speech and language therapist (*n* = 1); an undefined combination of AHPs and other healthcare professionals (*n* = 1); and undefined hospital-wide healthcare professionals likely to incorporate AHPs (*n* = 7). See Table 4 for the full study characteristics and Fig. 2 for the relevancy to the protocol’s inclusion criteria of all included studies. See Additional file 4 for the quality assessment of each individual study.

**Table 4:** Study characteristics

	***Country***	***Clinical Area***	***Population (P)***	***Intervention (I)***	***Study type /methods (S)***	***Outcomes (healthcare performance)***
**Anaby et al., 2015** [22]	Canada	Paediatrics	Health care professionals (Partial sample of AHP)	Knowledge translation implementation	Qualitative Research	Change in perception; intention for change; change in actual behaviour
*Bampton et al., 2012 [35]	Australia	Neurology	Physiotherapists	Delivering the therapy programme within an randomised control trial	Mixed Methods	Desire to be involved in another research project; positive and negative aspects of being involved in research
**Bottari et al., 2016** [23]	Canada	Neurology	Occupational therapists	Engagement in a knowledge translation toolkit programme	Qualitative Research	Development of a knowledge implementation toolkit for clinicians to use in practice
*Christensen et al., 2017 [24]	United States of America	Paediatrics	Physiotherapists	Implementation of a revised knowledge translation programme	Quasi-Experimental Studies	Development and compliance with local guidance; number of academic outputs and dissemination
**Dilworth et al., 2014** [25]	Australia	Oncology	Health care professionals (Partial sample of AHP)	Group clinical supervision for health care professionals trained to deliver a new psychosocial intervention as part of a step wedged randomised control trial	Qualitative Research	Impact of multi-disciplinary team and group clinical supervision on discourse of health professionals delivering psychosocial interventions
**Du Bois et al., 2005** [40]	Germany	Oncology	Health care professionals (Partial sample of AHP)	Hospitals participating in cooperative prospective randomised studies	Cross Sectional Studies	Survival rates; standard treatments received; higher proportions of patients ending up with optimal treatment
*Fary et al., 2015 [26]	Australia	Musculoskeletal physiotherapy	Physiotherapists	Enrolment in a randomised control trial which involved access to e-learning with follow up in a prospective cohort study following study completion	Randomised Controlled Trials	Self-reported confidence managing patients clinically; clinical vignettes as a proxy to measure processes of care
**Hadley-Barrows et al., 2017** [27]	United Kingdom	Musculoskeletal physiotherapy	Physiotherapists	Research facilitator role	Qualitative Research	Improved services and patient care
**Hébert-Croteau et al., 1999** [36]	Canada	Oncology	Health care professionals (Partial sample of AHP)	Hospitals participating in multi-centre trials	Cross Sectional Studies	Receiving treatment consistent with guidelines; speed of adoption of new interventions
**Kelley et al., 2012** [28]	Ireland	Multiple clinical areas	Occupational Therapists	Participatory action research cycle	Qualitative Research	Confidence and competence with applying research; behaviour change in vocational rehab and evidence based practice; effects on wider staff
*Kirby et al., 2020 [29]	United Kingdom	Respiratory	Paramedics	Participation in a randomised controlled trial	Mixed Methods	Changes in views and practice, engagement with research, professional identity, and professional competence
**Laliberte et al., 2005** [37]	United States of America	Oncology	Health care professionals (Partial sample of AHP)	Memberships in research networks	Cross Sectional Studies	Compliance with guidelines
**Lawford et al., 2019** [30]	Australia	Musculoskeletal physiotherapy	Physiotherapists	Participation in a randomised controlled trial	Qualitative Research	Willingness to embrace a different service delivery model following engagement
**Majumdar et al., 2008** [38]	United States of America	Cardiology	Health care professionals (Partial sample of AHP)	Hospitals participating in clinical trials	Cross Sectional Studies	Adherence with clinical guidelines
**Missiuna et al., 2013** [31]	Canada	Paediatrics	Occupational Therapists	‘Partnering 4 Change’ stakeholder collaboration	Qualitative Research	Perceptions, skills and confidence in new ways of working; clinical skills and confidence
**Naismith et al., 2011** [39]	United Kingdom	Oncology	Health care professionals (Partial sample of AHP)	Hospital participated in randomised control trial	Cross Sectional Studies	Development of new programmes; reduced resistance to change; supported evolution of practice; integration into daily departmental practice
**Nielsen et al., 2014** [32]	Australia	Musculoskeletal physiotherapy	Physiotherapists	Training to undertake a validated intervention within a randomised control trial	Qualitative Research	Positive and negative perceptions of engaging in a study; clinical skills and confidence; implementation of the approach into routine clinical practice
**Pons et al., 2010** [42]	Spain	Cardiology	Health care professionals (Partial sample of AHP)	Hospitals voluntarily participating in an external quality initiative	Cross Sectional Studies	Data from mortality and research outputs; measures of publications associated with the hospital; bibliometric measures of research outputs
**Rochon et al., 2011** [41]	Germany	Oncology	Health care professionals (Partial sample of AHP)	Hospital engagement in a study	Cross Sectional Studies	Outcome of newly diagnosed patients; adherence to treatment guidelines
*Salbach et al., 2010 [8]	Canada	Neurology	Physiotherapists	Self-reported research participation	Cross Sectional Studies	Association between participation in research with research use
**Tilson et al., 2014** [33]	United States of America	Multiple clinical areas	Physiotherapists	Participation in physical therapist-driven Education for Actionable Knowledge Translation (PEAK) programme	Mixed Methods	Impact of participation in project on self-reported evidence-based practice behaviour; integration of research into practice
*Webster et al., 2021 [34]	United Kingdom	Oncology	Therapeutic radiographers	Participation in a clinical trial quality assurance programme	Mixed Methods	Change in self-reported practice; change in wider clinical practice and departmental processes

*Papers highlighted as important are indicated with an asterisk

#### Importance

There were six papers of high importance [8, 24, 26, 29, 34, 35], and the other sixteen papers of low importance. Papers highlighted as important are starred in Table 4 and Table 5.

**Table 5:** Improvement assessment and identified mechanisms

*Study name*	*Importance*	*Level of study*	*Impact*	*Finding*	*Improvement identified*	*Mechanisms identified and extracted (coded)*
**Anaby et al., 2015** [22]	2	C	S	+	P	1.2a, 1.2b, 4.1
*Bampton et al., 2012 [35]	1	C	S	M +	P	1.2a, 1.2b, 2.1, 2.2, 2.3, 3
**Bottari et al., 2016** [23]	2	C	S	+	P	1.2.a, 1.2b, 3, 4.1
*Christensen et al., 2017 [24]	1	O	S	+	P	1.1, 1.2a, 2.1, 2.2, 3
**Dilworth et al., 2014** [25]	2	C	B	M +	P	1.2a, 1.2b
**Du Bois et al., 2005** [40]	2	O	B	+	HO	3,1.2b, 2.1
*Fary et al., 2015 [26]	1	C	S	+	P	1.2a; 1.2b; 2.2; 4.3
**Hadley-Barrows et al., 2017** [27]	2	O	B	+	P	1.2b
**Hébert-Croteau et al., 1999** [36]	2	O	S	+	P	1.2b, 2.1
**Kelley et al., 2012** [28]	2	C/O	S	+	P	1.2a, 5
*Kirby et al., 2020 [29]	1	C	S	M +	P	1.2a
**Laliberte et al., 2005** [37]	2	O	B	+	P	1.2b; 4.1; 4.3
**Lawford et al., 2019** [30]	2	C	S	+	P	1.2b
**Majumdar et al., 2008** [38]	2	O	S	+	P	1.2b
**Missiuna et al., 2013** [31]	2	O	S	+	P	1.2a, 1.2b, 3, 4.1, 5
**Naismith et al., 2011** [39]	2	O	S	+	P	-
**Nielsen et al., 2014** [32]	2	C	S	+	P	1.2a,1.2b, 2.1
**Pons et al., 2010** [42]	2	O	B	+	HO	-
**Rochon et al., 2011** [41]	2	O	B	+	HO	1.1; 1.2b; 2.1; 3
*Salbach et al., 2010 [8]	1	C	B	+	P	1.2a
**Tilson et al., 2014** [33]	2	C	B	+	P	1.2a, 1.2b, 3, 4.1
*Webster et al., 2021 [34]	1	C	B	M +	P	1.2a, 1.2b, 3, 4.1

Importance: 1 = High, 2 = Low; Level of study engagement: O = Organisational level, C = Clinician level; Impact: S = Specific, B = Broad; Finding: + Positive,—Negative, M Mixed, M + Mixed-positive, M- Mixed-negative; Improvement identified: P = Processes of care, HO = Health outcomes; Mechanisms identified and extracted: 1.1 Changes in the structure of institutions – improvements in infrastructure, 1.2 Changes in human capital, 1.2a Training/updating staff through research engagement leading to the acquisition and use of new skills, other gains in knowledge and changes in attitudes towards research and research findings, 1.2b Enhancement of group and individual behaviour including more rapid uptake of new treatments and greater likelihood of following clinical guidelines, and improved collaboration, establishment of expert teams, etc., 2.1

A more rigorous process of defining the standard of care for patients irrespective of their inclusion in the trial, 2.2 More close monitoring and support, 2.3 Early access to novel technologies, 3 Organisational mechanisms within health-care systems, 4.1 Linkage and exchange that improves the relevance of research and policy-makers'/managers'/clinicians' willingness to use it, 4.2 Academic Health Science Centres, teaching/research hospitals, 4.3 Research networks as an increasingly important mechanism, 5 Action research and participatory research as mechanisms that improve relevance, understanding of research and willingness to use research

*Papers highlighted as important are indicated with an asterisk

#### Level of study engagement

There were eleven studies which focused on research engagement at the clinician level [8, 22, 23, 25, 26, 29, 30, 32–35]. Ten studies focused on research engagement at the organisational level [24, 27, 31, 36–42], and one study referred to both [28].

#### Impact

Thirteen studies reported a specific impact; all of which showed positive findings in relation to healthcare performance, specifically processes of care [22–24, 26, 28–30, 32, 35, 36, 38, 39]. Nine studies reported a broad impact, all of which showed positive findings in relation to processes of care (*n* = 6) [8, 25, 27, 33, 34, 37] and health outcomes (*n *= 3) [40–42].

### Type of research engagement intervention

Ten studies were judged to meet the protocol’s inclusion criteria for intervention with clear engagement *in* research [8, 25, 29, 30, 32, 35, 39–42]. Examples of engagement in research within these studies included:

- being directly involved in delivering an intervention within a clinical research study;
- hospitals which participated at some stage in clinical research; and
- self-reported participation in research

Of these, 5 of these studies were judged to meet the protocol’s inclusion criteria for population [8, 29, 30, 32, 35] and of these only 2 papers were judged to meet the protocol’s inclusion criteria for study type to indicate an association between engagement in research and health outcomes [8, 29]. These two studies were judged to be of high importance. Both of these studies had mixed-positive [29] and positive [8] outcomes on processes of care with both specific and broad impacts respectively.

A further six studies were judged to meet the broadened inclusion criteria for intervention: research engagement with a mixed scenario of engagement *in* research and engagement *with* research [22, 24, 28, 31, 34, 37]. Examples of mixed scenarios of research engagement included:

- knowledge translation implementation including research activity such as scoping reviews, research implementation, reading research articles, and evidence-based learning
- knowledge translation programme including research studies, journal clubs, and Critically Appraised Topics
- participatory action research cycles
- hospitals which were involved in a research network group
- participation in a Partnering for Change stakeholder group to transform service delivery
- participation in a clinical trial quality assurance programme

Of these, five studies met the protocol’s inclusion criteria for population [22, 24, 28, 31, 34] and of these, only one met the criteria of study type [24] and therefore was evaluated to be of high importance. This study had positive findings in relation to processes of care which had a specific impact.

The remaining six studies were judged to meet the broadened inclusion criteria for intervention that described engagement *with* research [23, 26, 27, 33, 36, 38]. Examples of engagement with research included:

- knowledge translation toolkit programme
- engagement with a e-learning modules containing evidence base and clinical cases
- engagement with a research facilitator
- hospital implementation of guidelines as part of a clinical trial
- participation in education for actionable knowledge translation

Of these, four met the protocol’s inclusion criteria of population [23, 26, 27, 33] and of these, one met the inclusion criteria of study type [26]. This was judged to be of high quality and therefore of high importance due to the high relevance to the inclusion criteria. This study showed positive findings for processes of care with a specific impact.

### Studies reporting positive findings

Of the 22 included studies, one randomised controlled trial (high quality) [26], one quasi-experimental study (high quality) [24], and one cross-sectional study (low quality) [8] were judged to meet the protocol’s inclusion criteria for both population of interest and study type (see Table 3 for description of positive/negative/mixed classification). However, the degree of research engagement was questionable and was not explicitly described in two of the studies [24, 26]. Despite this, these three studies were identified to be of high importance because of the combined relevance to the protocol’s inclusion criteria for study type and overall quality, population, and intervention. All three studies reported a positive impact of research engagement on specific [24, 26] and broad [5] processes of care.

Four mixed methods studies were included and were judged to meet the protocol’s inclusion criteria for the population of interest [29, 33–35]. Only two studies used an intervention which would meet the protocol’s inclusion criteria of engagement in research [29, 35]. None of the four studies used a study type incorporated within the protocol’s inclusion criteria; however, these studies were included following the decision to broaden the inclusion criteria [29, 33–35]. Out of these four mixed methods studies, three studies were judged to be of high quality [29, 33, 35] and one was judged to be of low quality [34]. Based upon relevancy to the protocol’s inclusion criteria and quality of the studies, three studies were identified to be of high importance. These studies showed both mixed-positive findings with broad impact [34] and mixed-positive findings with specific impact [29, 35] for improving processes of care. The remaining study was evaluated to be of low importance and was classified to have positive findings with broad impact for improving processes of care [33].

Seven of the cross-sectional studies included only partial populations of AHPs, [36–42]. Four out of these seven cross-sectional studies were judged to meet the protocol’s inclusion criteria for the intervention in that the study made explicit reference to engagement *in* research [39–42]. Six out of the seven studies were graded to be of high quality [36–39, 41, 42] and one study was judged to be of low quality [40]. Due to the mixed population of health professionals and variation of the research engagement intervention, the seven studies were judged to be of low importance. Four of the studies had positive findings with regard to whether research engagement had a specific and broad impact and improved processes of care [36–39]. Furthermore, three of the studies had positive findings with regard to whether research engagement had a broad impact and improved health outcomes [40–42].

Six out of the eight qualitative studies were judged to meet the protocol’s inclusion criteria for the population of interest [23, 27, 28, 30–32], the remaining two studies having a partial population of AHP [22, 25]. Only two of these qualitative studies were judged to make explicit reference to engagement *in* research [25, 32]. Furthermore, the majority of the studies were classified to be of low quality [23, 25, 27, 28, 30, 31]. Thus, due to the methodological design and the variation in appropriate intervention, all eight qualitative studies were judged to be of low importance. All eight qualitative papers were positive with regard to whether research engagement improved specific [22, 23, 28, 30–32] and broad processes of care [25, 27].

### Mechanisms of the research engagement intervention

The most common mechanism (see Table 5 for full list of mechanisms and corresponding studies) identified through which healthcare performance improved across the included studies was that AHP research engagement may facilitate a ‘change in human capital’. This is in both enhancement of group and individual behaviour including more rapid uptake of new treatments and greater likelihood of following clinical guidelines (*n* = 16) [22, 23, 25–27, 30–32, 35–38, 40, 41] and training/updating staff through research engagement leading to the acquisition and use of new skills; and change in attitudes towards research and research findings (*n* = 13) [8, 22–26, 28, 29, 31–35]. Mechanisms of ‘improvements in the processes of care related to conducting a specific trial’ were commonly identified; these were a more rigorous process of defining the standard of care (*n* = 6) [24, 32, 35, 36, 40, 41] and closer monitoring and support (*n* = 3) [24, 26, 35].

Mechanisms of improvement were also identified at organisational and clinician levels.

‘Organisational mechanisms’ were identified in seven studies [23, 24, 31, 33–35, 40, 41]; these were a global category of conducting research to address known issues in the healthcare system, allowing AHPs time to conduct research and thus being an attractive organisation to work for, and conducting research to identify best performance targets and using research in quality improvement. Mechanisms of improvement related to ‘collaborative working between organisations, teams and individuals’ were identified in seven studies, specifically: linkage and exchange that improves the relevance of research and policy-makers’/managers’/clinicians’ willingness to use it (*n* = 6) [22, 23, 31, 33, 34, 37]; and research networks (*n* = 2) [26, 37]. Finally, mechanisms of improvement which were less commonly identified were ‘action and participatory research’ (*n* = 2) [28, 31] and ‘changes in the structures of institutions’ (*n* = 2) [24, 41].

## Discussion

This review supports the agenda for growing AHP research and innovation, and the findings generated have developed our understanding of the value of research engagement to assist AHP clinicians, managers, leaders, and academics to evaluate potential AHP research and innovation activities. This is a landmark systematic review as it provides essential robust information to inform debates, future research and practice. The findings indicate that AHP research engagement appears related to positive findings in improvements to processes of care, but falls short of providing evidence of the degree of effectiveness. Whilst we are unable discuss definitively the degree of effectiveness in traditional terms, the tentative information we have collected from this review supports existing policy which calls for an AHP workforce that is research engaged and the infrastructure to support this [11]. The studies included allow us to open the debate and discuss the value of research engagement more widely to benefit stakeholders of health and social care systems, and present the implications and recommendations for future research designs and evaluation approaches in academia and in practice.

The review findings report both broad and specific impacts in relation to improvements to processes of care, by using Hanney et al.’s matrix [7]. Examples of the broad impacts included: improved services and patient care in general [27], association between participation in research and using research in practice [8], and impact on self-reported evidence-based practice behaviour and implementation of research into practice [33]. Examples of specific impacts included: the development of clinical guidance [24], number of academic outputs [24], self-reported confidence in clinical patient management [26, 31, 32], and embracing a different service delivery model [30, 32]. These findings correlate to the findings of Boaz et al. who similarly identified positive findings in the wider population of healthcare professionals [6].

Amongst the generally positive findings and impact of AHP research engagement, there are a small number of studies which highlight a more balanced picture of where AHP research engagement may be associated with mixed-positive findings [25, 29, 34, 35] (i.e. where the findings were mostly positive with a small degree of negative findings in comparison). The studies which showed mixed-positive findings were mostly qualitative evaluations of research engagement, such as AHP perceptions of the impact on clinicians willingness to follow research protocols which deliver interventions different to usual care, and the additional responsibilities on clinical AHPs to be engaged in a trial [35]. The included studies suggest positive and mixed-positive findings, therefore a positive reporting bias amongst these studies is acknowledged. These findings further demonstrate that recommendations to practice cannot be firmly made based on this review.

Mechanisms that acted as levers to instigate a relationship between research engagement and improved healthcare performance were explored using the framework from the previous review by Hanney et al. [7]. It is acknowledged that this was a secondary aim of our review and that exploration of these mechanisms was not the primary aim of the studies included. However, during the review process it was highlighted that consideration of these mechanisms has a particular importance and significance for future research and practice. Two most common mechanisms have been identified: 1) ‘Changes in human capital’ and 2) ‘Organisational mechanisms within healthcare settings’. The mechanism ‘Changes in human capital’ may relate to building research capability, for example facilitating changes in the knowledge, skills, education and attitudes of staff through research engagement. This mechanism may lead to more rapid uptake of new treatments and greater likelihood of following clinical guidelines [7]. ‘Organisational’ mechanisms relate to the influence of allowing time for AHPs to be research engaged, being an attractive organisation to work for, and using research in improvement projects [7]. In addition, this also related to a wider mechanism of collaborative approaches between organisations, teams, and individuals that improves research relevancy and willingness to use research findings [7].

Identifying the workforce and organisational mechanisms which facilitate the realisation of research engagement benefits for impact on service quality and care is crucial. Specifically in England, in the UK, a multi-professional practice-based research capabilities framework has recently been commissioned within Health Education England’s workforce transformation remit, to support the development of research-related skills and confidence in an incremental progression across the career span. This will build on the basic regulatory requirement of research design and process knowledge specified in AHP preregistration curricula [43] with the focus on driving evidence-based quality improvement for all health and care service sectors (publication forthcoming). In addition, clearer definitions of such mechanisms are essential for systematic facilitation of strategic approaches that may be implemented within organisations. Mechanisms that support research engagement may already be available and achievable within current organisational systems and processes, provided that there is a more equitable and proportionate investment commitment to facilitate this proactively for the AHP workforce. To secure the requisite investment, the value of AHP research engagement needs to be ‘sold’ to service providers and commissioners in the currency of the organisations’ priorities for workforce transformation, safety culture, and quality of service user experience [44, 45].

The process of conducting this review with a specific lens on the evidence for AHP research engagement has highlighted the need for a number of significant methodological refinements in future studies specifically around the population, intervention, and outcomes. This variation in the literature suggests that the conventions for reporting are still insufficiently specific, and the community has not yet managed to adopt and consistently use a systematic approach. This is problematic and not necessarily unique to AHP. It invites a standardisation exercise in the future, with a more robust and systematic means of appropriately capturing the value and impact of research engagement on healthcare performance, in addition to other outcome measures. In particular, in the context of research engagement by multidisciplinary teams and for all collaborative research initiatives, it is strongly recommended that all relevant contributions and attributions should be explicated [46].

### AHP population

This review provides a specific focus on AHPs as opposed to incorporating the wider healthcare disciplines. Whilst it is very encouraging to see explicit evidence of AHP research activities in this review sample, further clarity of reporting is recommended about the AHP participants, especially within multi-disciplinary teams and services. In some of the included studies, for example rehabilitation teams in specified clinical specialisms, the AHP contingent of the workforce is implicit only. Of the included studies, only five of the registered AHPs were named (occupational therapists, physiotherapists, speech and language therapists, paramedics, and radiographers). Whilst AHPs collectively are regulated by common competency standards [47], it is acknowledged that the respective disciplines are at difference stages of self-efficacy in terms of success and confidence in research engagement. This is directly attributed to historical interdisciplinary disparities in their access to investment in research leadership, with consequent respective ongoing needs for targeted support. This agenda is being addressed by the recently launched HEE AHP Research and Innovation Strategy [11], to secure more equitable access and progress in supporting context and infrastructure. More detailed reporting of the specific AHP disciplines in future studies would enable synthesis of findings to increase the collective evidence for value and impact. In addition, this would help to demonstrate differential needs for greater support where needed. The widespread adoption of common evaluation approaches and tools is strongly advocated, to facilitate collective future benchmarking and progress monitoring at three distinctive levels – organisational, team, and the individual practitioner [48–50].

### Research engagement interventions

The research team addressed the challenge of the complex and multifaceted definitions of research engagement and subsequent varied terminology that was identified in the current published evidence base. The loose distinctions between research or service improvement activities, in comparison with knowledge transfer, implementation, and evidence-based continuing professional development were problematic. The broadened inclusion criteria therefore maximised inclusivity and a shift was made to encompass engagement *with* research as well as *in* research, as previously defined by Hanney et al., so that all publications that could potentially contribute relevant insights would be examined. It is highly recommended that future studies could benefit from more standardised common reporting using the EQUATOR suite of guidelines [51]. Prospective designs and the grouping of different types of research engagement activities are also recommended. This recommendation is in line with the universal call for explicit support strategies to facilitate implementation of research into practice, as highlighted in the joint position statement issued in 2021 by the Professors of Allied Health embedded in Health Services in Australia [3]. A structured framework has been developed for the evaluation of a suite of proposed strategies to support research engagement [52].

Based on the papers included in this review, the concept ‘research engagement’ goes beyond the active involvement, of the traditional and narrow definition of, ‘research’; and rather incorporates a broad range of activities that includes awareness, understanding, and contributions that have the potential to benefit knowledge exchange, learning and trust between different professional groups, organisations, and communities. The concept of engagement *in* and *with* research included in this review may also be limited in teasing out the complexities. The broader concept of ‘research engagement’ may better reflect the wider initiatives set out in policy to support the research and innovation agenda, for example research, innovation, quality improvement, leadership, service improvement, research delivery, and scholarly activities [11]. This warrants the development of more detailed descriptions and understandings about the diversity of activities and contexts of research.

### Measurement of the value of research engagement

A range of relevant and appropriate approaches to evaluation of outcomes will be an essential component of future study protocols, to generate the robust evidence sources needed by managers of AHP services to support the agenda that research engagement by the workforce may credibly lead to improved healthcare performance. The findings from this review will inform the design of prospective future research, to more specifically and appropriately reflect and evaluate the impact and value of AHP research engagement. In line with the four domains addressed in the HEE Research and Innovation Strategy [11], the protocols of future studies need to differentiate more precisely between outcomes in terms of capability building of skills and careers for individuals, versus capacity building for evidence-based practice, and implementation of research in routine practice by the wider workforce. These refinements in specificity will further assist greater clarity and understanding in communicating the concept of research engagement, and allow for detailed and collective evaluation of activities, for example guided by the Medical Research Council (MRC) framework for developing and evaluating complex interventions [53].

## Strengths and limitations

The original intention of this review was to adopt the methodology [6, 7] by Hanney et al. and Boaz et al. to generate a contemporary re-focused update of the impact of AHP engagement in research. Due to the initial paucity of papers that fit the protocol inclusion criteria, the decision to broaden the protocol’s inclusion criteria maximised inclusivity to study type, research engagement intervention, and mixed populations of clinicians (rather than solely AHPs). As a result, the overall quality of evidence was suboptimal. Furthermore, sub-group analysis to evaluate the importance of mechanisms was not possible due to the heterogeneity of studies. Accordingly, we are making clear recommendations for future research design with cautious recommendations in relation to the implications for organisations and services.

The strengths of the review methods include the use of a multi-database search and that we broadened the inclusion criteria from our protocol which resulted in the highest possible recall. Dual paper screening was conducted at all stages with high inter-rater reliability indicated with a Kappa score (a statistical measure of inter-rater reliability). The frameworks and data extraction forms were tested to ensure relevancy to the study aims and parity between the researchers, with ongoing discussions with the research team to ensure consistency and consensus. To facilitate this decision-making process, a pre-tested set of criteria was used to enhance repeatability. However, despite the use of the criteria, there is always aspects of subjectivity in this type of decision-making process.

The heterogeneity of studies, and limitations in level of indirectness and quality may have called for alternative review methods. Although outside the scope of this review, a series of reviews to scope the available literature may have been useful to conduct a contemporary evaluation of research engagement terminology to gain greater conceptual clarity. Furthermore, a more recent taxonomy and range of mechanisms and outcomes relating to AHPs specifically may have been facilitated by a series of alternative research questions. Due to the use of a previous review search strategy, it is possible there may be reduced recall as the subject domain and corresponding terms set may need updating.

The research team represent a mix of allied health professions (speech and language therapy and physiotherapy) working in the UK with a range of research experience (pre-doctoral to professorial) and roles (some solely academic or strategic, some more clinical). It is recognised that this means the team are invested in research as a worthwhile endeavour, but are also aware of the challenges and reality of this. Our allied health and academic backgrounds have allowed our interpretations of the findings from the review to be appropriately set within the broader context. Equally however, we acknowledge that our professional backgrounds may question our biases towards the benefits of allied health research activity. The research team are based in the UK, and this review used a definition of AHPs consistent with Health Education England. We recognise this may introduce geographical bias in this review, despite including international papers and setting the findings within a broader context.

## Implications

This review supports existing policy that aims to drive the agenda to accelerate the growth of AHP research and innovation. By providing a synthesis of the current evidence base that explicates the value of AHP research engagement, this information can support AHP managers and leaders whose roles involve implementation of the recently published HEE AHP Research and Innovation strategy [11]. The review findings also provide a springboard for future research investment and coordinate a consistent and coherent approach to research designs in future. However as reported here, the current sample included a range of study designs. One feasible way to prospectively evaluate the impact of local research engagement could be to align a pre- and post-study within current primary AHP research activities. This may also perpetuate more specific reporting of variables such as AHP profession, clinical areas of practice, research engagement intervention types, and outcomes, with sub-group analysis of important mechanisms or instigators of change. Those study designs could better enable AHP leaders to capture the broader impact on the local workforce, service delivery, and clinical outcomes. This in turn would actively contribute to the wider AHP agenda by adding to the knowledge base.

On a larger scale, cross-sectional studies are advocated for the future, as being more appropriate to demonstrate effect by comparing engaged and non-engaged workforce groups across organisations [54]. It is acknowledged, however, that such studies will have limitations of defining these populations due the complexity in defining research engagement (as discussed in the subsection above) and responder bias will be likely. A shift in expectation for pre- and post-studies of research engagement within clinical trials is advocated. In the UK in particular, the existing infrastructure of Clinical Research Networks could facilitate this as a standard approach, to more efficiently capture and reflect the collective impact of research engagement in Portfolio studies (clinical research studies that are supported by NIHR Clinical Research Network in England, UK). In summary, studies are urgently needed that expressly address this research question to evaluate the impact of research engagement by AHPs, not only as a secondary outcome.

As identified, there is lack of clarity around mechanisms. Deeper examination of the importance of the varying mechanisms in this review was not possible, nor was the primary focus of the review. This prompts a recommendation for further exploratory research into the mechanisms which indicate a link between research engagement and healthcare performance. Based on the studies that reported positive findings in this review, ‘changes in human capacity’ and ‘organisational’ mechanisms were the most common mechanisms and may be of particular interest and importance when considering the types of interventions that might support research engagement. These mechanisms indicate some starting points when considering intervention approaches to enhance research engagement, which have also been evaluated by other researchers, for example AHP research training and academic education [55], clinical-academic roles [56] and career pathways [57], dedicated research time [58], the role of research facilitators/brokers [59], and research leadership and social influence [60]. It is recommended that in future there is a co-ordinated approach to the implementation of such interventions, and a collective approach to evaluation to demonstrate change. Due to the inconsistencies in outcomes and unknowns of effect, the value of qualitative research could be used to explore the unexpected impacts of research engagement, similar to a review in 2019 [12] but with a specific focus on AHPs, and the mechanisms that link research engagement and the associated impacts.

## Conclusion

The findings of this review have affirmed that the current published sources comprise a generally positive, albeit limited, evidence base for AHP research engagement with broad and specific impacts. The review also identifies the most common mechanisms which may link research engagement with improvements to processes of care. These workforce and organisational mechanisms correspond to the cultural and contextual factors highlighted by the HEE AHP Research Strategy [11] and which may be important for future exploration and evaluation.

Recognition of the value, importance, and reputation of AHP research engagement is wholly dependent upon the development and implementation of agreed evaluation approaches and metrics. Our review has highlighted the need for greater specificity in future study protocols. Specifically, this includes the transparency of AHP workforce participation in uni- and multi-professional contexts, research engagement activities, and outcomes. In addition, our review has demonstrated the priority need for explicit consensus on the most relevant and appropriate indicators of value and impact of AHP research engagement.

Recommendations are made for approaches which would enable more transparency and could explicitly capture and evaluate the impact and value of clinicians who are research engaged. It is more time-critical than ever before to develop and refine more standardised methodologies, frameworks and infrastructure to promote AHP research engagement evaluation. Suggestions have been made in which AHP managers, clinicians, and leaders, and researchers may contribute to the needed evidence to demonstrate the value of research engagement for clinical services and the collective AHP workforce. That collective evidence base is needed to support the strategic leverage for research engagement to be embedded in national agendas [9, 11] by AHP managers and leaders who are calling for sustainable investment and facilitation of AHP research and innovation.

## Supplementary Information

**Additional file 1.** Example search strategy adapted from Boaz et al. 2015.

**Additional file 2.** Example data extraction form.

**Additional file 3.** Pre-defined coding framework for the mechanisms in research-active settings through which healthcare improved (Hanney et al. 2013, page 79, box 4).

**Additional file 4.** Quality assessment of included studies.

## Data Availability

All data generated or analysed during this study are included in this published article and its additional information files.

## Abbreviations

AHP: Allied Health Professional/s
HEE: Health Education England
NHS: National Health Service

## Footnotes

1 See methods section for full descriptions and examples of activities captured by each term.

2 The Allied Health Professions (AHP) in England include the following named disciplines: Art Therapists, Drama Therapists, Music Therapists, Dietitians, Occupational Therapists, Operating Department Practitioners, Orthoptists, Osteopaths, Paramedics, Physiotherapists, Podiatrists, Prosthetists and Orthotists, Diagnostic and Therapeutic Radiographers, and Speech and Language Therapists.

3 See methods section for the precise, narrow definition of ‘value’ used in this review.
