# Supplementary material for: The value of allied health professional research engagement on healthcare performance: a systematic review
Source: BMC Health Serv Res. 2023 Jul 18;23:766. doi: 10.1186/s12913-023-09555-9 (PMC10355072; doi:10.1186/s12913-023-09555-9)
Supplement: Supplementary file 1 — Additional file 1. Example search strategy adapted from Boaz et al. 2015. [file 12913_2023_9555_MOESM1_ESM.docx]

## Additional file 1

## Example search strategy adapted from Boaz et al. 2015

| **Database: Ovid MEDLINE(R) ALL** |
| --- |
| 1 ((engag$ adj2 research$) or (engag$ adj2 trial?) or (engag$ adj2 case stud$) or (engag$ adj2 clinical stud$) or (engag$ adj2 experimental therap$) or (engag$ adj2 RCT?) or (engag$ adj2 randomi?ed controlled trial?) or (engag$ adj4 clinical trial?) or (participat$ adj4 research$) or (participat$ adj4 trial?) or (participat$ adj4 case stud$) or (participat$ adj4 clinical stud$) or (participat$ adj4 experimental therap$) or (participat $ adj4 RCT?) or (participat$ adj2 randomi?ed controlled trial?) or (participat$ adj4 clinical trial?) or (involv$ adj2 research$) or (involv$ adj2 trial?) or (involv$ adj2 case stud$) or (involv$ adj2 clinical stud$) or (involv$ adj2 experimental therap$) or involv$adj2 RCT? or (involv$ adj2 randomi?ed controlled trial?) or (involv$ adj4 clinical trial?) or (interact$ adj2 research$) or (interact$ adj2 trial?) or (interact$ adj2 case stud$) or (interact$ adj2 clinical stud$) or (interact$ adj2 experimental therap$) or interact$adj2 RCT? or (interact$ adj2 randomi?ed controlled trial?) or (interact$ adj4 clinical trial?) or (tak$ part adj3 research$) or (tak$ part adj5 trial?) or (tak$ part adj3 case stud$) or (tak$ part adj3 clinical stud$) or (tak$ part adj3 experimental therap$) or (tak1art adj3 RCT?) or (tak$ part adj2 randomi?ed controlled trial?) or (tak$ part adj4 clinical trial?) or (initiat$ adj2 research$) or (initiat$ adj2 trial?) or (initiat$ adj2 case stud$) or (initiat$ adj2 clinical stud$) or (initiat$ adj2 experimental therap$) or (initiat$ adj2 RCT?) or (initiat$ adj2 randomi?ed controlled trial?) or (initiat$ adj4 clinical trial?) or (follow$ adj2 research$) or (follow$ adj2 trial?) or (follow$ adj2 case stud$) or (follow$ adj2 clinical stud$) or (follow$ adj2 experimental therap$) or (follow$ adj2 RCT?) or (follow$ adj2 randomi?ed controlled trial?) or (follow$ adj4 clinical trial?) or (introduc$ adj2 research$) or (introduc$ adj2 trial?) or (introduc$ adj2 case stud$) or (introduc$ adj2 clinical stud$) or (introduc$ adj2 experimental therap$) or introduc$adj2 RCT? or (introduc$ adj2 randomi?ed controlled trial?) or (introduc$ adj4 clinical trial?) or (conduct$ adj2 research$) or (conduct$ adj2 trial?) or (conduct$ adj2 case stud$) or (conduct$ adj2 clinical stud$) or (conduct$ adj2 experimental therap$) or (conduct$ adj2 RCT?) or (conduct$ adj2 randomi?ed controlled trial?) or (conduct$ adj4 clinical trial?) or learning organi?ation? or research intensive organi?ation? or academic medical centre? or academic medical center? or academic health science centre? or academic health science center? or research network? or research collaboration? or study hospital? or teaching research facilities or trial hospital? or veterans health administration).ti,ab. (226705)  2 ((improve$ or influence$ or determine$ or affect$ or effect$ or increase$ or decrease$ or declines$ or diminish$ or weake$ or worse$ or benefi$ or impact$ or better or worse or greater or lesser or lower or higher or evaluat$ or compar$) adj5 (performance or (patient$ adj4 outcome?) or process quality or process assessment? or (health care adj4 outcome?) or (healthcare adj4 outcome?) or (clinical adj4 outcome?) or (quality adj4 care) or (compar$ adj4 outcome?) or (patient$ adj4 mortality) or (routine adj clinical practice) or (mortality adj4 outcome$) or organi?ational process$ or organi?ational determinant$ or organi?ational characteristic? or organi?ational innovation? or organi?ational culture or organi? ational support or (clinical adj2 care) or treatment outcome or (adhere$ adj4 guideline?) or ("use$" adj4 guideline?) or clinical practi?e or patient$satisfaction)).ti,ab. (710962)  3 ((practice adj4 change?) or (service adj4 change?) or organi?ational change? or treatment change? or prescri$ change?).ti,ab. (26321)  4 (allied health practit* or allied health clinician or allied health personnel or art therap* or drama therap* or music therap* or chiropod* or podiatr* or dietitian or dietician or dietetic or occupational therap* or operating department pract* or orthopt* or osteopath* or paramedic* or physiotherapy* or physical therap* or prosthet* or orthoti* or radiograph* or speech language therap* or speech language patholog*).ti,ab. (370026)  5 1 and 4 (5496)  6 2 or 3 (734130)  7 5 and 6 (760)  8 7 (760)  9 limit 8 to english language (754)  10 limited 8 to yr="2012-2021") (574)  11 9 and 10 (568) |
