# Supplementary material for: The value of allied health professional research engagement on healthcare performance: a systematic review
Source: BMC Health Serv Res. 2023 Jul 18;23:766. doi: 10.1186/s12913-023-09555-9 (PMC10355072; doi:10.1186/s12913-023-09555-9)
Supplement: Supplementary file 2 — Additional file 2. Example data extraction form. [file 12913_2023_9555_MOESM2_ESM.docx]

## Additional file 2

## Example data extraction form

| **Authors** | **Date** | **Paper Title** | **Country** | **Research Question** | **Clinical Area** | **Population (P)** | **Intervention (I)** | | **Study design/methods (S)** | **Outcomes (all)** | **Outcomes (research engagement)** |
| --- | --- | --- | --- | --- | --- | --- | --- | --- | --- | --- | --- |
|  |  |  |  |  |  |  |  | |  |  |  |
| **(Example data extraction form continued)** | | | | | | | | | | | |
| **P-Score** | **I-Score** | **S-Score** | **Overall quality** | **Importance** | **Degree of intentionality** | **Level of study engagement** | **Impact** | **Finding** | **Improvement identified** | **Free comments** | **Mechanisms identified and extracted (coded)** |
| Classification of indirectness (1-10 scale): applicability of included studies to the protocol inclusion criteria | | | High/low: quality based on quality assessment | High/low: Based on integrated assessment of study quality and study type, followed by indirectness of population and intervention | Intervention, by-product, or network paper | Clinician or organisational level of engagement | Broad or specific | Positive, negative, mixed, mixed-positive, mixed-negative | Processes of care, or healthcare outcomes |  | According to the pre-defined coding framework (additional file 3) |
