# Supplementary material for: The value of allied health professional research engagement on healthcare performance: a systematic review
Source: BMC Health Serv Res. 2023 Jul 18;23:766. doi: 10.1186/s12913-023-09555-9 (PMC10355072; doi:10.1186/s12913-023-09555-9)
Supplement: Supplementary file 3 — Additional file 3. Pre-defined coding framework for the mechanisms in research-active settings through which healthcare improved (Hanney et al. 2013, page 79, box 4). [file 12913_2023_9555_MOESM3_ESM.docx]

## Additional file 3

## Pre-defined coding framework for the mechanisms in research-active settings through which healthcare improved (Hanney et al. 2013, page 79, box 4)

| **Code** | **Mechanism** |
| --- | --- |
| **1**  **1.1**  **1.2**  **1.2a**  **1.2b** | **Absorptive capacity: this is most relevant for wider adoption of research in institutions**  *Changes in the structure of institutions – improvements in infrastructure*   - Attributes of the setting in which care is delivered, such as accommodation, equipment and personnel, which are brought in to perform research-related activities and may remain in place after the research is completed, for example in the UK the capital spend on the Biomedical Research Centres which resulted in new co-located research and clinical facilities   Changes in human capital  *Training/updating staff through research engagement* leading to the acquisition and use of new skills, other gains in knowledge and changes in attitudes towards research and research findings  *Enhancement of group and individual behaviour* including more rapid uptake of new treatments and greater likelihood of following clinical guidelines, and improved collaboration, establishment of expert teams, etc. |
| **2**  **2.1**  **2.2**  **2.3** | **Improvements in the processes of care related to conducting a specific trial**  *A more rigorous process of defining the standard of care for patients irrespective of their inclusion in the trial*  *More close monitoring and support*  *Early access to novel technologies* |
| **3** | **Organisational mechanisms within health-care systems**  For example, in the VA, where the whole organisation uses research to improve health care in various and integrated ways, including conducting research to address known issues in the health-care system, allowing physicians time to conduct research and thus being an attractive organisation to work for, conducting research to identify best performance targets to set, using research in QI, etc. |
| **4**  **4.1**  **4.2**  **4.3** | **Collaborative approaches between organisations, teams and individuals as a mechanism**  *Linkage and exchange that improves the relevance of research and policy-makers'/managers'/clinicians' willingness to use it*  *Academic Health Science Centres, teaching/research hospitals*  *Research networks as an increasingly important mechanism* |
| **5** | **Action research and participatory research as mechanisms that improve relevance, understanding of**  **research and willingness to use research** |
