# Supplementary material for: The value of allied health professional research engagement on healthcare performance: a systematic review
Source: BMC Health Serv Res. 2023 Jul 18;23:766. doi: 10.1186/s12913-023-09555-9 (PMC10355072; doi:10.1186/s12913-023-09555-9)
Supplement: Supplementary file 4 — Additional file 4. Quality assessment of included studies. [file 12913_2023_9555_MOESM4_ESM.docx]

## Additional file 4

## Quality assessment of included studies

| **Joanna Briggs Institute (JBI) Critical Appraisal Tools checklist questions** | | | | | | | | | | | | | |
| --- | --- | --- | --- | --- | --- | --- | --- | --- | --- | --- | --- | --- | --- |
| **Randomised control trials** | | | | | | | | | | | | | |
|  | **1. Was true randomisation used for assignment of participants to treatment groups?** | **2. Was allocation to treatment groups concealed?** | **3. Were treatment groups similar at the baseline?** | **4. Were participants blind to treatment assignment?** | **5. Were those delivering treatment blind to treatment assignment?** | **6. Were outcomes assessors blind to treatment assignment?** | **7. Were treatment groups treated identically other than the intervention of interest?** | **8. Was follow up complete and if not, were differences between groups in terms of their follow up adequately described and analyzed?** | **9. Were participants analyzed in the groups to which they were randomised?** | **10. Were outcomes measured in the same way for treatment groups?** | **11. Were outcomes measured in a reliable way?** | **12. Was appropriate statistical analysis used?** | **13. Was the trial design appropriate, and any deviations from the standard RCT design accounted for in the conduct and analysis of the trial?** |
| Fary et al, 2015 | Yes | No | Yes | Yes | No | Yes | Yes | Yes | Yes | Yes | Yes | Yes | Yes |
| **Quasi-experimental** | | | | | | | | | | | | | |
|  | **1. Is it clear in the study what is the ‘cause’ and what is the ‘effect’ (i.e. there is no confusion about which variable comes first)?** | **2. Were the participants included in any comparisons similar?** | **3. Were the participants included in any comparisons receiving similar treatment/care, other than the exposure or intervention of interest?** | **4. Was there a control group?** | **5. Were there multiple measurements of the outcome both pre and post the intervention/exposure?** | **6. Was follow up complete and if not, were differences between groups in terms of their follow up adequately described and analyzed?** | **7. Were the outcomes of participants included in any comparisons measured in the same way?** | **8. Were outcomes measured in a reliable way?** | **9. Was appropriate statistical analysis used?** |  |  |  |  |
| Christensen et al, 2017 | Yes | Yes | Unsure | No | No | Unsure | Yes | Yes | No |  |  |  |  |
| **Cross sectional** | | | | | | | | | | | | | |
|  | **1. Were the criteria for inclusion in the sample clearly defined?** | **2. Were the study subjects and the setting described in detail?** | **3. Was the exposure measured in a valid and reliable way?** | **4. Were objective, standard criteria used for measurement of the condition?** | **5. Were confounding factors identified?** | **6. Were strategies to deal with confounding factors stated?** | **7. Were the outcomes measured in a valid and reliable way?** | **8. Was appropriate statistical analysis used?** |  |  |  |  |  |
| Hébert-Croteau et al, 1999 | Yes | Yes | Yes | Yes | Yes | Yes | Yes | Yes |  |  |  |  |  |
| Laliberte et al, 2005 | Yes | Yes | Yes | Yes | Yes | Yes | Yes | Yes |  |  |  |  |  |
| Majumdar et al 2008 | Yes | Yes | Yes | Yes | Yes | Yes | Yes | Yes |  |  |  |  |  |
| Pons et al, 2010 | Yes | Yes | Yes | Yes | Yes | Yes | Yes | Yes |  |  |  |  |  |
| Rochon et al, 2011 | Yes | Yes | Yes | Yes | Yes | Yes | Yes | Yes |  |  |  |  |  |
| Du Bois et al, 2005 | Yes | Yes | Yes | Yes | Yes | Unsure | Yes | Yes |  |  |  |  |  |
| Salbach et al, 2010 | Yes | Yes | Unsure | Unsure | Unsure | Unsure | Yes | Unsure |  |  |  |  |  |
| Naismith et al, 2011 | No | No | No | Unclear | No | No | No | No |  |  |  |  |  |
| **Qualitative** | | | | | | | | | | | | | |
|  | **1. Is there congruity between the stated philosophical perspective and the research methodology?** | **2. Is there congruity between the research methodology and the research question or objectives?** | **3. Is there congruity between the research methodology and the methods used to collect data?** | **4. Is there congruity between the research methodology and the representation and analysis of data?** | **5. Is there congruity between the research methodology and the interpretation of results?** | **6. Is there a statement locating the researcher culturally or theoretically?** | **7. Is the influence of the researcher on the research, and vice- versa, addressed?** | **8. Are participants, and their voices, adequately represented?** | **9. Is the research ethical according to current criteria or, for recent studies, and is there evidence of ethical approval by an appropriate body?** | **10. Do the conclusions drawn in the research report flow from the analysis, or interpretation, of the data?** |  |  |  |
| Anaby 2015 | NA | Yes | Yes | Yes | Yes | No | No | Yes | Yes | Yes |  |  |  |
| Bottari et al, 2016 | Unclear | Unclear | Unclear | Unclear | Unclear | No | No | Unclear | Yes | Unclear |  |  |  |
| Dilworth et al, 2014 | No | Yes | Unclear | Unclear | Unclear | No | No | No | No | Unclear |  |  |  |
| Hadley-Barrows et al, 2017 | Unclear | Unclear | Unclear | Unclear | Unclear | No | Unclear | Unclear | Unclear | Unclear |  |  |  |
| Kelley et al, 2013 | Unclear | Unclear | Unclear | Unclear | Unclear | No | No | Unclear | Unclear | Unclear |  |  |  |
| Lawford et al, 2019 | Yes | Yes | Yes | Yes | Yes | Unclear | Yes | Yes | Yes | Yes |  |  |  |
| Missiuna et al, 2013 | Unclear | Unclear | Unclear | Unclear | Unclear | No | Unclear | Unclear | Unclear | Unclear |  |  |  |
| Nielsen et al, 2014 | Unclear | Yes | Yes | Yes | Yes | No | No | Yes | Yes | Yes |  |  |  |
| **Mixed Methods Appraisal Tool (http://mixedmethodsappraisaltoolpublic.pbworks.com/w/page/24607821/FrontPage)** | | | | | | | | | | | | | |
| **Mixed methods** | | | | | | | | | | | | | |
|  | **S1. Are there clear research questions?** | **S2. Do the collected data allow to address the research questions?** | **1.1. Is the qualitative approach appropriate to answer the research question?** | **1.2. Are the qualitative data collection methods adequate to address the research question?** | **1.3. Are the findings adequately derived from the data?** | **1.4. Is the interpretation of results sufficiently substantiated by data?** | **1.5. Is there coherence between qualitative data sources, collection, analysis and interpretation?** | **2.1. Is randomization appropriately performed?** | **2.2. Are the groups comparable at baseline?** | **2.3. Are there complete outcome data?** | **2.4. Are outcome assessors blinded to the intervention provided?** | **2.5 Did the participants adhere to the assigned intervention?** | **3.1. Are the participants representative of the target population?** |
| Bampton et al, 2012 | Yes | Yes | Yes | Yes | Yes | Yes | Yes | NA | NA | NA | NA | NA | NA |
| Tilson et al, 2014 | Yes | Yes | Yes | Yes | Can't tell | Can't tell | Yes | NA | NA | NA | NA | NA | NA |
| Kirby et al, 2020 | Yes | Yes | Yes | Yes | Yes | Yes | Yes | NA | NA | NA | NA | NA | Survey yes, interview no |
| Webster et al, 2021 | Yes | Yes | Yes | No | Can't tell | No | No | NA | NA | NA | NA | NA | NA |
| **Mixed methods (continued)** | | | | | | | | | | | | | |
|  | **3.2. Are measurements appropriate regarding both the outcome and intervention (or exposure)?** | **3.3. Are there complete outcome data?** | **3.4. Are the confounders accounted for in the design and analysis?** | **3.5. During the study period, is the intervention administered (or exposure occurred) as intended?** | **4.1. Is the sampling strategy relevant to address the research question?** | **4.2. Is the sample representative of the target population?** | **4.3. Are the measurements appropriate?** | **4.4. Is the risk of nonresponse bias low?** | **4.5. Is the statistical analysis appropriate to answer the research question?** | **5.1. Is there an adequate rationale for using a mixed methods design to address the research question?** | **5.2. Are the different components of the study effectively integrated to answer the research question?** | **5.3. Are the outputs of the integration of qualitative and quantitative components adequately interpreted?** | **5.4. Are divergences and inconsistencies between quantitative and qualitative results adequately addressed?** |
| Bampton et al, 2012 | NA | NA | NA | NA | Yes | Yes | Yes | Can't tell | Yes | Yes | Yes | Yes | Yes |
| Tilson et al, 2014 | NA | NA | NA | NA | Yes | Can't tell | Yes | Yes | Can't tell | Yes | Yes | Can't tell | No |
| Kirby et al, 2020 | Yes | Yes | No | Unsure | Yes | Yes | Yes | No | Yes | Yes | Yes | Yes | Yes |
| Webster et al, 2021 | NA | NA | NA | NA | Can't tell | Can't tell | No | Can't tell | No | Can't tell | No | No | No |
| **Mixed methods (continued)** | | | | | | | | | | | | | |
|  | **5.5. Do the different components of the study adhere to the quality criteria of each tradition of the methods involved?** |  |  |  |  |  |  |  |  |  |  |  |  |
| Bampton et al, 2012 | Yes |  |  |  |  |  |  |  |  |  |  |  |  |
| Tilson et al, 2014 | Can't tell |  |  |  |  |  |  |  |  |  |  |  |  |
| Kirby et al, 2020 | Yes |  |  |  |  |  |  |  |  |  |  |  |  |
| Webster et al, 2021 | No |  |  |  |  |  |  |  |  |  |  |  |  |
